# Failure to fail: Fear of retribution or a response to neglecting the learner?

**DOI:** 10.1111/medu.14796

**Published:** 2022-03-15

**Authors:** Joy R. Rudland, Sarah C. Rennie

**Affiliations:** ^1^ Educational Development and Staff Support University of Otago Wellington New Zealand; ^2^ Education Unit University of Otago Wellington New Zealand

## Abstract

Rudland and Rennie comment on Swails et al.'s observation that staff are failing fewer learners, offering reflection on the impact neglecting a learner may have on educator decisions if the failure to fail phenomenon is considered.

1

In this issue Swails et al.^1^ give another angle on the ‘failure to fail’ debate. ‘Failure to fail’ has been well documented through a systematic review^2^ and is characterised by educators feeling unwilling and/or unprepared to report unsatisfactory trainee performance. Swails et al. indicate two additional factors at play; firstly, they found that junior members of faculty are less likely to give what they term low performance evaluation (LPE) compared to their more senior colleagues, and secondly, they found that LPEs have decreased over the past 15 years; fewer learners are failing.

LPEs have decreased over the past 15 years; fewer learners are failing.

The decrease in the number of learners with LPEs is curious, although competency‐based education with clear outcomes and supportive teaching (including midpoint feedback) may offer better routes for learner improvement. However, the authors suggest that junior staff, who do the majority of evaluations, fail to fail learners due to fear of detrimental consequences in the form of student evaluations rather than improved performance of the learner. Other explanations may also be present. Swails et al. allude to changing work patterns that limit the time faculty spends with individual learners. Healthcare systems appear busier now than 15 years ago with increasingly sick, elderly comorbid patients. With clinicians working to capacity there may be less room for quality education.^3^ This may relate to learner neglect, a relatively new concept that can be defined as a lack of care, attention and/or input given to the educational process of a learner.^4^ Learner neglect may include the educator being emotionally and physically unavailable, failing to recognise learner individuality and failure to promote social adaptation.^4^

With clinicians working to capacity there may be less room for quality education.

While learner neglect is applied to the learner, it is also useful to consider the impact that neglecting a learner may have on the educator. There is a danger that an educator may, consciously or unconsciously, feel inadequate in the supervision, education and evaluation of the learner.^2^ Research into medical error highlights the complex role of guilt and shame in medical education.^5^ A supervisor who does not perform the educational role as well as they expect may feel persecutory guilt. Persecutory guilt arises when accountability demands are not met^6^ and an individual feels remorse.^7^ In response to persecutory guilt, a supervisor may exhibit reparative behaviours,^8^ which often occur after an individual has transgressed in their obligations. They include benefit to another person (a learner in this case) in an attempt to make amends^9^ or comply with demands.^6^ Learner neglect may be due to external pressures placed on busy clinicians and/or poor support on how to supervise students or to adequately evaluate the learner performance. The learner may be of secondary importance when the healthcare of individuals or populations is under threat.

While learner neglect is applied to the learner, it is also useful to consider the impact that neglecting a learner may have on the educator.

Learner neglect may therefore inadvertently be a factor in ‘failing to fail’ as well as the other factors stated. There is a need for a greater understanding why some learners are neglected. Equally some supervisors may need support in how to ‘care’ appropriately for learners, particularly those who are felt to be failing. There is a need for clear guidance on what ‘fail’ means—particularly moving away from the negative connotations associated with the word. It is also necessary to support supervisors to better manage learners' reactions to failure^10^ and to know how to remediate a learner to enable and facilitate their growth.

Decisions regarding failing a student may also be closely related to the rise and ‘fear’ of litigation.^11^ If a supervisor has neglected the learner, then a learner who ‘fails’ may be more likely to make a claim against poor educational practice. It may be easier to pass a learner than to acknowledge or expose deficits in one's own supervisory performance,^2^ especially for those seeking tenured posts. Poor supervisory oversight may be due to neglect but also to a lack of clear guidance on the process of how to fail a student in a positive, supportive manner that facilitates the learner's growth and development.^2^ In addition, how a diligent supervisor is supported when failing a learner may be unclear and potentially onerous.

It may be easier to pass a learner than to acknowledge deficits in one's own supervisory performance.

Swails et al.'s hypothesis that junior faculty may be hesitant to document substandard student performance due to fear of negative consequences, particularly poor teaching evaluations, supports findings elsewhere.^2^, ^12^ Teaching evaluations are an important component of assessment for promotion, selection for teaching awards, and other advancement and leadership opportunities. The finding that increased experience in completing LPEs does not influence the awarding of LPEs could support the focus on securing tenure over the need for accurate student evaluations. However, it is unclear why these factors may have had more impact recently. Changes over time may relate to the specific programme and context; for example, greater competition for tenure or changes in tenure regulations at the institution leading to the award of fewer LPEs.

Not having the longitudinal data on the demographics of the evaluators hinders the interpretation of the results seen, for example, were there more full professors grading students in 2007 than in 2021? It could be expected that the number of women evaluators has increased over the time period studied, however, with the significant difference seen between men and women, with more women providing LPEs, a rise not a fall may be expected in the number over time. The finding that women grade harder than men is fascinating and warrants further exploration of the reasons. It is unclear what the interaction is between gender and seniority.

This commentary is littered with terms of self‐conscious emotions such as fear and guilt. Emotions have been under‐researched in health professional education; it may be useful to develop a linguistic framework to clarify terms of self‐conscious emotions in health professional education, as much for the educator as for the learner. The real challenge may be in enabling and supporting supervisors to engage meaningfully with learners, to ensure the learner is not neglected, thus allowing a supervisor to genuinely ‘fail’ a learner with confidence.

The real challenge may be in enabling and supporting supervisors to engage meaningfully with learners, to ensure the learner is not neglected.
